# Multidrug-Resistant Bacteria in Southeastern Austria

**DOI:** 10.3201/eid1308.070283

**Published:** 2007-08

**Authors:** Alexandra Badura, Gebhard Feierl, Harald H. Kessler, Andrea Grisold, Lilian Masoud, Ute Wagner-Eibel, Egon Marth

**Affiliations:** * Medical University of Graz, Graz, Austria

**Keywords:** Antibiotic resistance, extended-spectrum beta-lactamases, multidrug-resistant bacteria, letter

**To the Editor:** In many parts of the world, the proportions of methicillin-resistant *Staphylococcus aureus* (MRSA), vancomycin-resistant enterococci, and extended-spectrum β-lactamase (ESBL)–producing organisms in the family *Enterobacteriaceae* have increased remarkably during recent years (*1*). However, proportions of antimicrobial drug resistance vary substantially at national and regional levels. We describe antimicrobial drug resistance data for hospitalized patients and outpatients in southeast Austria.

A total of 690,967 clinical samples were collected from hospitalized patients and outpatients and analyzed at the microbiology laboratory of the Medical University of Graz during 1997–2006. Selected for resistance surveillance were nonduplicate isolates of *S. aureus, Enterococcus faecium, E. faecalis, Escherichia coli,* and *Klebsiella* spp. Antibiotic susceptibilities were determined by using disk diffusion and the VITEK2 system (bioMérieux, Marcy l’Etoile, France) with specific susceptibility test cards. Etest (AB Biodisk, Solna, Sweden) was used to confirm results. Test results were interpreted according to the recommendations of the Clinical and Laboratory Standards Institute (*2*).

During the study period, the proportion of patients with MRSA remained stable (2.5%–4.9%) (Figure, panel A). The prevalence of MRSA among invasive *S. aureus* isolates ranges between 0.5% and 44.4% in European countries and has increased in recent years (*3*). We found MRSA predominantly in samples from hospitalized patients (median 72.1%); however, the incidence of community-acquired MRSA increased slightly during recent years, similar to that of other central European countries (*4*). Vancomycin resistance was not noted during the study period; however, 4 vancomycin-intermediate MRSA isolates were noted in 2004, 2005, and 2006, in concordance with the sporadic occurrence of MRSA with intermediate susceptibility to glycopeptides recently reported for other European countries (*3*).

The percentage of patients with vancomycin-resistant *E. faecium* and *E. faecalis* was low (median 0.4%) (Figure, panel A). In total, 10 *E. faecium* and 4 *E. faecalis* isolates with resistance to vancomycin were reported; most were from hospitalized patients. As in most European countries, human infections due to glycopeptide-resistant enterococci remain rare in Austria, although a high proportion of glycopeptide-resistant *E. faecium* was reported recently from animals used in food production (*5*).

Among *E. coli* isolates, no ESBL producers were noted in 1997. From 1998 through 2002, proportions of ESBL-producing *E. coli* were 0.06%–0.13%, which corresponds to 3–6 isolates per year. A subsequent increase of ESBL-producing *E. coli* isolates was noted, from 19 (0.3%) in 2003 to 148 (2.4%) in 2006 (Figure, panel B). Most (67%) ESBL-producing isolates found during 2003–2006 originated from community-acquired urinary tract infections. Resistance of *E. coli* to carbapenems was not reported during the study period. Among *Klebsiella* spp. isolates, 2 (0.2%) ESBL producers were observed during 1997. From 1998 through 2004, the prevalence of ESBLs among *Klebsiella* spp. ranged between 0.6% and 1.6%. In 2005 and 2006, the rate of ESBL-producing *Klebsiella* spp. increased to 3.8% (44 isolates) and 4.5% (55 isolates), respectively, and originated mainly from intensive care units (Figure, panel B). In 2005, a single *Klebsiella pneumoniae* isolate showed reduced susceptibility to imipenem (MIC 2 μg/mL) and to meropenem (MIC 4 μg/mL) and resistance to ertapenem (MIC >16 μg/mL). Nevertheless, production of ESBL by *Enterobacteriaceae* organisms is still rare in southeast Austria compared with other European countries (*6*). However, a dramatic increase of ESBL-producing *E. coli* and *Klebsiella* spp. has been observed during recent years.

The increase of ESBL-producing *E. coli* isolates in outpatients with urinary tract infections leads to serious treatment problems. Results from a recent study indicate that the increase of ESBL-producers in southeast Austria is caused mainly by the emergence of CTX-M–type ESBLs, which are increasingly being isolated from outpatients (*7*). The *K. pneumoniae* isolate found in 2005 represents the first ESBL-producing isolate not susceptible to carbapenems reported from Austria. Development of resistance to carbapenems in *Enterobacteriaceae* organisms has been reported increasingly, which substantially limits treatment options for persons with multidrug-resistant gram-negative infections (*8*).

Our data show insignificant changes in prevalence of MRSA and vancomycin-resistant enterococci in southeast Austria during the past decade but an alarming increase of multidrug-resistant ESBL-producing *E. coli* and *Klebsiella* spp. isolates in recent years. Detection of an ESBL-producing *K. pneumoniae* isolate with reduced susceptibility to carbapenems shows that pathogens with new mechanisms of resistance are emerging in this region.

**Figure:**
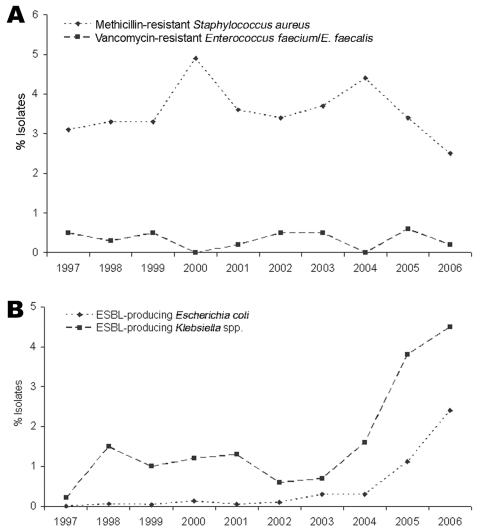
A) Proportion of methicillin resistance in *Staphylococcus aureus* and vancomycin resistance in *Enterococcus faecium* and *E. faecalis* in southeastern Austria, 1997–2006. B) Proportion of extended-spectrum β-lactamase–producing (ESBL) *Escherichia coli* and *Klebsiella* spp. in southeastern Austria, 1997–2006.
